# Replication Attempt: “Effect of BMAP-28 Antimicrobial Peptides on Leishmania Major Promastigote and Amastigote Growth: Role of Leishmanolysin in Parasite Survival”

**DOI:** 10.1371/journal.pone.0114614

**Published:** 2014-12-17

**Authors:** Elizabeth Iorns, William Gunn, Jessey Erath, Ana Rodriguez, Jian Zhou, Michael Benzinou, The Reproducibility Initiative

**Affiliations:** 1 The Reproducibility Initiative, c/o Science Exchange, Palo Alto, California, United States of America; 2 Anti-Infectives Screening Core Facility, Department of Microbiology, New York University School of Medicine, New York, New York, United States of America; 3 LifeTein LLC, Hillsborough, New Jersey, United States of America; Tulane University, United States of America

## Abstract

This study describes an attempt to replicate experiments from the paper “Effect of BMAP-28 Antimicrobial Peptides on *Leishmania major* Promastigote and Amastigote Growth: Role of Leishmanolysin in Parasite Survival,” which was submitted to the Reproducibility Initiative for independent validation. The cathelicidin bovine myeloid antimicrobial peptide 28 (BMAP-28) and its isomers were previously shown to have potent antiparasitic activity against *Leishmania major.* We tested the effectiveness of L-BMAP-28 and two of its isomers, the D-amino acid form (D-BMAP-28) and the retro-inverso form (RI-BMAP-28), in both unamidated and amidated forms, as anti-leishmanial agents against *Leishmania major* promastigotes *in vitro*. We observed that L-BMAP-28, as well as its D and RI isomers, demonstrate anti-leishmanial activity against *L. major* promastigotes *in vitro*. The inhibitory effect was lower than what was seen in the original study. At 2 µM of amidated peptides, the viability was 94%, 36%, and 66% with L-, D- and RI-peptides, versus 57%, 6%, and 18% in the original study.

## Introduction

Most scientific advancement occurs not through blockbuster papers that definitively solve a problem, but through careful and incremental progress, building on prior work. However, recent work by the pharmaceutical companies Bayer Healthcare [1] and Amgen [2] suggest that ∼75% of academic preclinical biomedical studies may not be reproducible. This highlights a significant problem that needs to be addressed to enable scientific progress. Nosek *et al*. [3] showed that this problem stems from publishing norms that incentivize novel positive findings. To combat these norms, the Reproducibility Initiative (www.reproducibilityinitiative.org) was created to promote a culture of reproducibility in academic research by identifying and rewarding reproducible research [4]. Reproducible work is identified through replication of key experimental results by independent professional research organizations using the Science Exchange network (www.scienceexchange.com).

The study “Effect of BMAP-28 Antimicrobial Peptides on Leishmania major Promastigote and Amastigote Growth: Role of Leishmanolysin in Parasite Survival” [5] was submitted by its authors to the Reproducibility Initiative for independent assessment.

Leishmaniasis is a serious disease that affects up to 20 million people globally [6]. Resistance to current therapies is a growing problem that limits effective treatment [7], [8]. The identification of BMAP-28 isomers as potent anti-leishmanials is therefore highly significant and represents an important experimental observation to independently validate. The original study [5] reported that the cathelicidin bovine myeloid antimicrobial peptide 28 (L-BMAP-28) and two of its isomers, the D-amino acid form (D-BMAP-28) and the retro-inverso form (RI-BMAP-28), had potent antiparasitic activity against *Leishmania major*, the parasite responsible for leishmaniasis.

The aim of this study was to determine whether the *in vitro* effect of BMAP-28 and its isomers against *L. major* promastigotes could be replicated by independent labs. Because it was not reported in the original study, we originally used unamidated BMAP-28 peptides. After observing that these peptides had inhibitory activity only at high concentrations, we communicated with the authors and clarified that they had used amidated peptides in their original experiments, thus we also evaluated amidated peptides. In addition, we performed a dose-response curve to gather more detailed data than the two doses used in the original article.

## Materials and Methods

### Peptide synthesis

Peptide synthesis was conducted by LifeTein (https://www.scienceexchange.com/facilities/lifetein). BMAP-28 (GGLRSLGRKILRAWKKYGPIIVPIIRIG and GGLRSLGRKILRAWKKYGPIIVPIIRI-NH_2_) isomers (all L amino acids, all D amino acids, retro-inverso amino acids) were chemically synthesized using Fmoc (9-fluorenylmethoxy carbonyl) chemistry. The peptide chains were synthesized from the carboxyl terminus to the Glycine amino terminus onto resin. The resin was incubated with dichloromethane (DCM) for 30 minutes and then washed with dimethylformamide (DMF) three times. Fmoc-protecting groups at the amino terminus were deprotected with an alkaline buffer and then washed with DMF three times to remove the deprotection buffer. The second amino acid was Fmoc-Ile-OH coupled to the first amino acid and then DMF cleaned. After each coupling, the peptide was ninhydrin tested and the coupling and washing steps repeated until the crude peptide was fully synthesized. For amidated peptides, Rink amide resin was used in solid phase peptide synthesis to prepare peptide amides utilizing Fmoc-protected amino acids. The completed peptides were cleaved from the Rink resin under acid conditions. The crude peptides were then diethyl ether precipitated, drained and washed. The peptides were isolated and purified by high-performance liquid chromatography (HPLC). Fractions of greater than 95% purity were used for the investigation. The purity and molecular weight of the respective peptides were confirmed by matrix-assisted laser desorption ionization (MALDI)-time of flight mass spectrometry.

### Promastigote viability assay

The promastigote viability assays were conducted by the Anti-Infectives Screening Core Facility at New York University School of Medicine (https://www.scienceexchange.com/facilities/anti-infectives-screening-and-insectary-core-facilities-nyu). All assays were performed by investigators blinded to the reagents under study. 500 µM stocks of peptides were made by resuspending 4 mg of each peptide (M.W. 3131.92) in 2.554 mL of distilled sterile water. Each peptide was tested in triplicate three independent times at 50 µM, 25 µM, 12.5 µM, 6.25 µM, 3.125 µM, 1.5625 µM, 0.78125 µM, and 0.390625 µM. *L. major* strain MHOM/SN/74/SD (ATCC, PRA-384) was used in these studies. These cells were routinely cultured at 26°C in M-199 medium (Gibco, Grand Island, NY), supplemented with 10% heat inactivated fetal calf serum (Hyclone, Logan) and Pen/Strep (Gibco, Grand Island, NY). Mid-log phase promastigotes at concentrations of 5×10^6^ per well were seeded in 96-well tissue culture plates. Heat-killed parasites, prepared by incubation at 99°C for 30 minutes, served as positive controls for the viability assays. Peptides were incubated with the cells for 4 hours at 26°C. A 20 µL volume of PrestoBlue (Life Technologies, Carlsbad, CA) was added to each well and incubated at 37°C for 3 hours. Fluorescence at excitation 530 nm and 590 nm emission wavelengths was measured using a plate reader (VICTOR, Perkin Elmer, US). Background fluorescence from media alone controls was subtracted from each reading and promastigote viability was expressed as a percentage of the untreated control.

Research resources were reported in accordance with the following guidelines: http://www.force11.org/node/4433.

### Statistical method

An independent-samples t-test was conducted to compare promastigote viability in peptide treated and untreated conditions. A Fisher combined probability test was used to combine the original and replication studies values into a single combined study value for each peptide and peptide concentration. To provide a standardized measure of the effect we used cohen's *d.*

## Results

**Figure 1:**
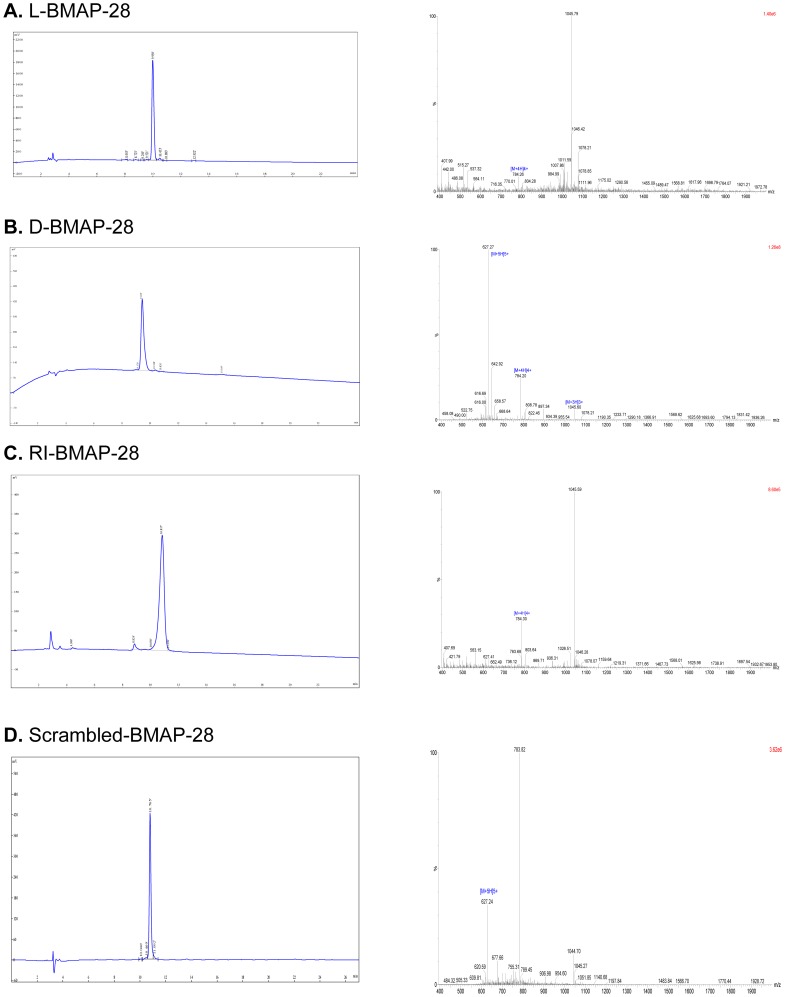
HPLC profile and mass spectrum of synthesized unmodified BMAP-28 peptides. The peptides (Sequence: GGLRSLGRKILRAWKKYGPIIVPIIRIG; M.W: 3131.92; Formula: C147H252N44O31) were isolated and purified by high-performance liquid chromatography (HPLC) to greater than 95% purity. The purity and molecular weight of the respective peptides were confirmed by matrix-assisted laser desorption ionization (MALDI)-time of flight mass spectrometry. Left panels: HPLC profile, right panels: mass spectrum. A) L-BMAP-28, purity 95.61%; B) D-BMAP-28, purity 96.67%; C) RI-BMAP-28, purity 95.62%; D) Scrambled-BMAP-28, purity 95.34%.

To independently test the anti-leishmanial activities of the BMAP-28 variants, unmodified peptide (L-BMAP-28), the retro-inverso isoform (RI-BMAP-28) and the D- amino acid isoform (D-BMAP-28) were synthesized using methods that matched those reported in the original study [5] as closely as possible. The peptides were purified to greater than 95% purity and the purity and molecular weight of the respective peptides were confirmed by matrix-assisted laser desorption ionization (MALDI)-time of flight mass spectrometry (Fig. 1).

**Figure 2:**
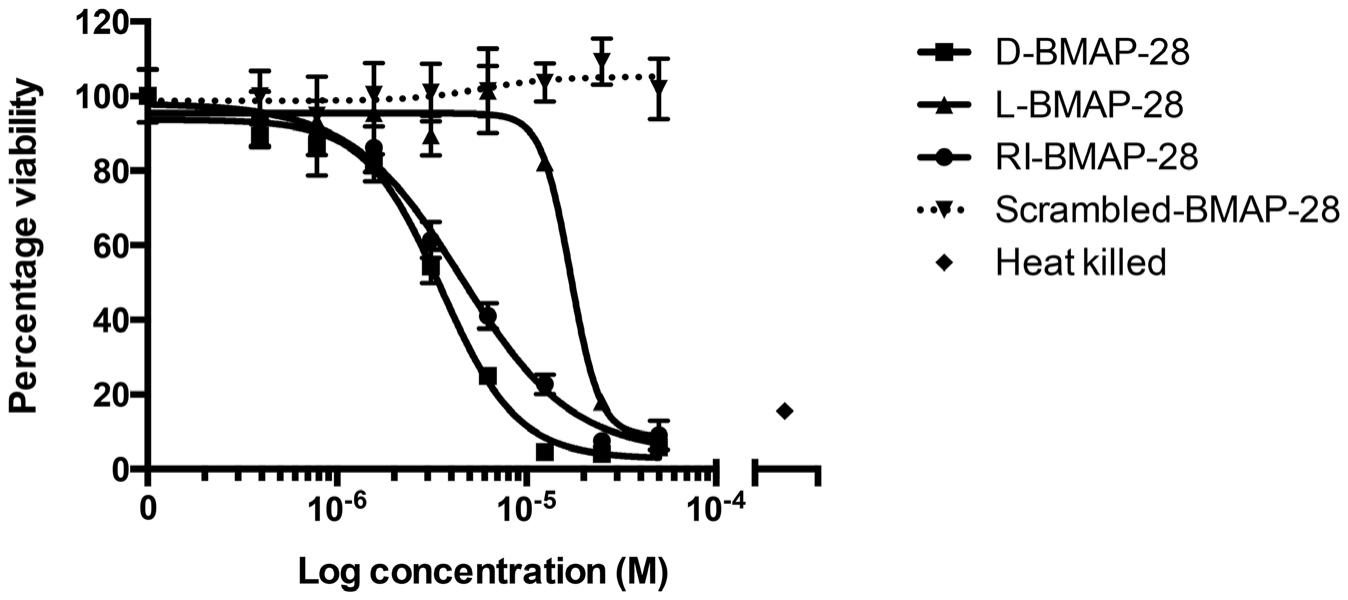
Promastigote viability assay of *Leishmania major* when treated with unmodified BMAP-28 variants. *L. major* MHOM/SN/74/SD strain was treated with L-, RI- and D-BMAP-28 peptides for 4 hours. Viability was expressed as a percentage of untreated control cells. Three complete biological replicates were performed and the standard errors are shown.

The synthesized unmodified peptides were then used to examine anti-leishmanial activity in *in vitro* promastigote viability assays, performed using methods that matched those reported in the original study [5] as closely as possible. Full experimental protocols were written based on the methods reported in the original paper and these were shared with the original study authors for comment prior to the assays being conducted. In the original study [5], only two concentrations of each peptide were used (0.5 µM and 2 µM). In this study, a range of peptide concentrations that spanned those in the original paper was used to create a dose-response curve (Fig. 2). Promastigote viability was expressed as a percentage of the untreated control. L-, RI- and D-BMAP-28 unmodified peptides all significantly reduced promastigote viability but they required high concentrations to achieve this. The IC_50_ values were 17.1 µM, 4.6 µM and 3.6 µM respectively. Of the three unmodified BMAP-28 variant forms, the D- form of the peptide was the most potent, followed by the RI-form, with the L-form showing the least potency. This order matched the order observed in the original study [5].

The percentage viability at 0.5 µM or 2 µM of each BMAP-28 peptide was calculated from the dose response curves performed (S1 Data) to enable comparison with the results presented in Fig. 1A of Lynn *et al*. [5]. We found that promastigote viability was reduced to 95%, 77% and 73% of untreated control when treated with 2 µM L-, RI- and D-BMAP-28, respectively. In contrast, the original study [5] found that promastigote viability was reduced to 57%, 18% and 6% (Table 1) when treated with 2 µM L-, RI- and D-BMAP-28, respectively.

**Table 1:** Promastigote viability assay of *Leishmania major* when treated with 0.5 µM or 2 µM BMAP-28 variants.

					95% Confidence Intervals		
Study	Treatment	% Viability (mean)	SD	d Effect Size	Lower	Upper	Unpaired t-test (two-tailed p-value)
Original	2 µM D-BMAP-28	5.50	4.33	37.80	35.80	39.80	0.0001
	0.5 µM D-BMAP-28	68.75	21.22	2.55	−7.25	12.35	0.063
	2 µM RI-BMAP-28	17.50	20.78	6.88	−2.72	16.48	0.0023
	0.5 µM RI-BMAP-28	103.00	4.33	−1.20	−3.20	0.80	0.3
	2 µM L-BMAP-28	56.75	24.25	3.09	−8.11	14.29	0.037
	0.5 µM L-BMAP-28	103.25	9.53	−0.59	−4.99	3.81	0.53
	Heat killed	8.25	0.43	-	-	-	-
	Untreated	100.00	0.00	-	-	-	-
Replication	2 µM D-BMAP-28-NH_2_	36.09	3.67	8.58	2.62	14.54	0.001
	0.5 µM D-BMAP-28-NH_2_	94.85	2.12	0.71	−5.09	6.51	0.52
	2 µM RI-BMAP-28-NH_2_	66.32	4.90	4.38	−1.76	10.53	0.012
	0.5 µM RI-BMAP-28-NH_2_	97.09	3.39	0.39	−5.53	6.32	0.71
	2 µM L-BMAP-28-NH_2_	94.15	1.72	0.81	−4.96	6.58	0.46
	0.5 µM L-BMAP-28-NH_2_	95.55	1.88	0.62	−5.16	6.40	0.57
	Heat killed	15.54	3.00	-	-	-	-
	Untreated	100.00	12.37	-	-	-	-
Combined	2 µM D-BMAP-28-NH_2_	20.80	4.00	16.67	13.98	19.36	1.70E–06
	0.5 µM D-BMAP-28-NH_2_	81.80	11.67	2.14	−2.69	6.96	0.14
	2 µM RI-BMAP-28-NH_2_	41.91	12.84	6.31	1.11	11.52	0.0003
	0.5 µM RI-BMAP-28-NH_2_	100.04	3.86	−0.01	−2.67	2.65	0.54
	2 µM L-BMAP-28-NH_2_	75.45	12.98	2.65	−2.61	7.90	0.086
	0.5 µM L-BMAP-28-NH_2_	99.40	5.70	0.11	−2.96	3.18	0.6664

*L. major* MHOM/SN/74/SD strain was treated with L-, RI- and D-BMAP-28 peptides for 4 hours. Viability was expressed as a percentage of untreated control cells. Three complete biological replicates were performed and the mean and standard deviation are shown. The percentage viability at 0.5 µM or 2 µM of each BMAP-28 peptide was calculated from the dose response curve performed (replication). The percentage viability at 0.5 µM or 2 µM of each BMAP-28 peptide was determined from the bar graph reported in Fig. 1A of Lynn *et al*. [5] (original). Unpaired t-tests untreated vs treated indicated significance.

**Figure 3:**
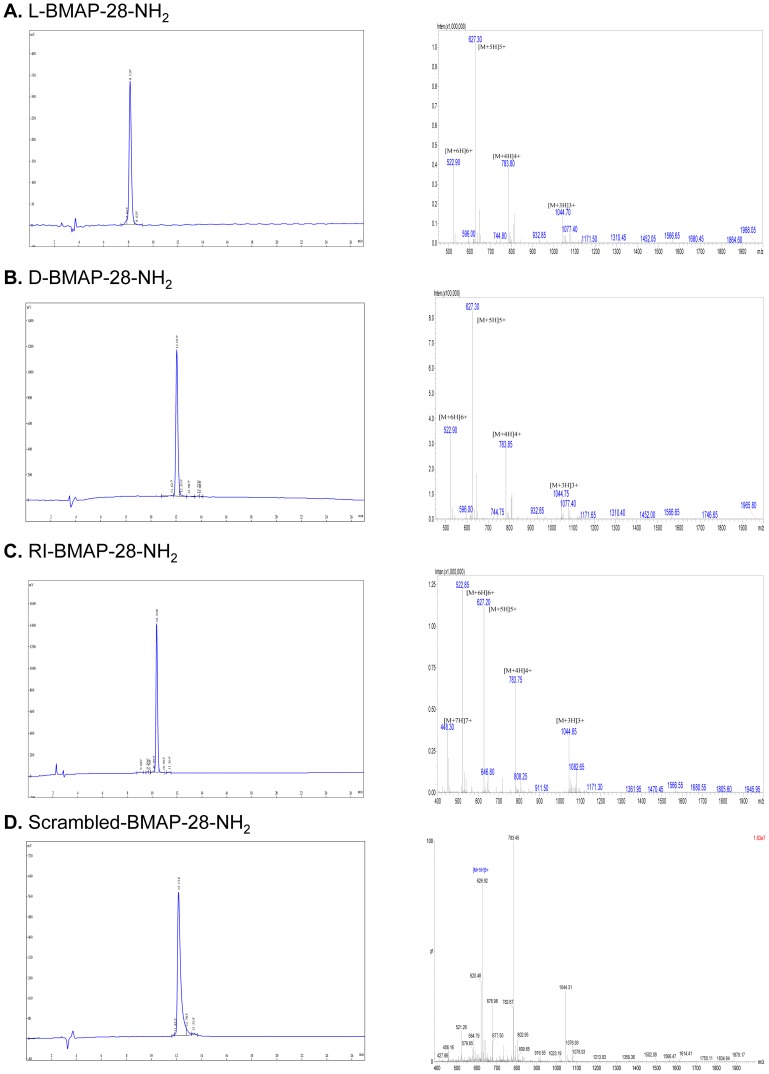
HPLC profile and mass spectrum of synthesized amidated BMAP-28 peptides. The peptides (Sequence: GGLRSLGRKILRAWKKYGPIIVPIIRI-NH_2_; M.W: 3131.92; Formula: C147H252N44O31) were isolated and purified by high-performance liquid chromatography (HPLC) to greater than 95% purity. The purity and molecular weight of the respective peptides were confirmed by matrix-assisted laser desorption ionization (MALDI)-time of flight mass spectrometry. Left panels: HPLC profile, right panels: mass spectrum. A) L-BMAP-28-NH_2_, purity 95.64%; B) D-BMAP-28-NH_2_, purity 95.35%; C) RI-BMAP-28-NH_2_, purity 95.85%; D) Scrambled-BMAP-28-NH_2_, purity 95.19%.

The differing potency of the BMAP-28 peptides in the promastigote viability assay was shared with the original study authors and it was determined that the peptides used in the original study had been modified by amidation. To compare the potency of amidated BMAP-28 peptides to unamidated, we synthesized amidated L-, D-, and RI- BMAP-28-NH_2_ peptides. The amidated peptides were purified to greater than 95%, and the purity and molecular weight of the respective peptides were confirmed by matrix-assisted laser desorption ionization (MALDI)-time of flight mass spectrometry (Fig. 3).

**Figure 4:**
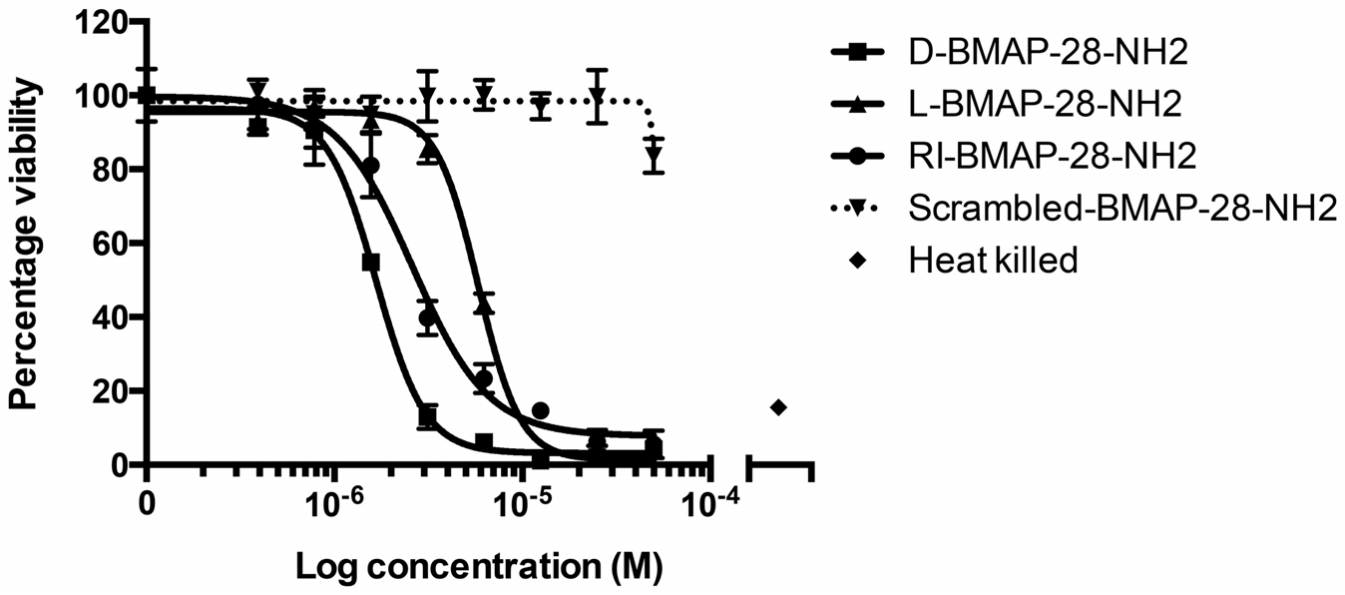
Promastigote viability assay of *Leishmania major* when treated with amidated BMAP-28 variants. *L. major* MHOM/SN/74/SD strain was treated with L-, RI- and D-BMAP-28 peptides for 4 hours. Viability was expressed as a percentage of untreated control cells. Three complete biological replicates were performed and the standard errors are shown.

The synthesized amidated peptides were then used to examine anti-leishmanial activity in *in vitro* promastigote viability assays (Fig. 4). Promastigote viability was expressed as a percentage of the untreated control. L-, RI-, and D-BMAP-28- NH_2_ peptides all significantly reduced promastigote viability (with IC_50_ values of 5.8 µM, 2.6 µM and 1.7 µM respectively). Of the three BMAP-28-NH_2_ variant forms, the D- form of the peptide was again the most potent, followed by the RI-form, with the L-form showing the least potency, the same order of potency observed for the unmodified peptides and in the original study [5].

The percentage viability at 0.5 µM or 2 µM of each BMAP-28 peptide was again calculated from the dose response curves performed (Table 1, S1 Data) to enable comparison with the results presented in Fig. 1A of Lynn *et al*. [5]. We found that promastigote viability was reduced to 94%, 66% and 36% of untreated control when treated with 2 µM L-, RI- and D-BMAP-28-NH_2_, respectively. The original study [5] found that promastigote viability was reduced to 57%, 18% and 6% (Table 1) when treated with 2 µM L-, RI- and D-BMAP-28, respectively. There was no overlap in the 95% confidence intervals around these values between the original study and our results, meaning that the results we obtained were significantly more conservative regarding the potential effectiveness of the peptides on promastigote viability. There are many reasons this replication may have found a 2-fold difference in the amount of peptide required to achieve a similar level of activity. Quantitation and dilution of the peptide could have yielded different absolute amount of peptide from the original experiment. The differences in the history of the L. *major* strain can lead to differential responses via changes in clonal lineages affecting things such as expression of proteases. Additionally, differences in how the assay was performed could alter results. For example, protein binding elements in the fetal calf serum can lead to experimental variability.

**Figure 5:**
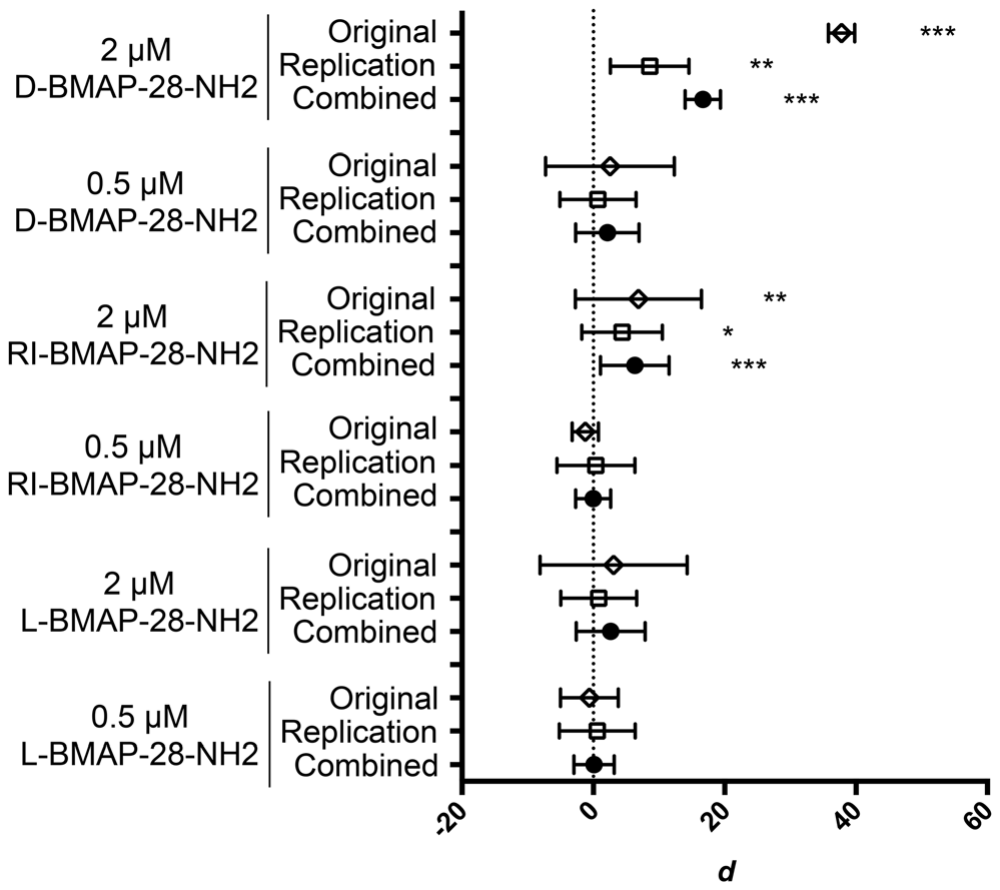
Cohen's d, 95% confidence intervals of the original and replication studies and their combination. Unpaired t-tests untreated vs treated indicated significance, where *p<0.05, **p<0.005, ***p<0.0005. The combined study p-values were generated using Fisher's combined probability test. The Forest plot was generated using GraphPad Prism version 6.

We conducted a meta-analysis of the original and replication studies to evaluate all the existing evidence for the effect (Table 1 and Fig. 5). To provide a standardized measure of the effect we used cohen's *d.* Combining the two datasets resulted in no significant change in the overall effect when treated with 2 µM L-BMAP-28 but the confidence interval (CI) and the significance of the test was decreased (combined d = 2.65, 95% CI [−2.61–7.90], p = 0.087 vs original d = 3.09, 95% CI [−8.11–14.29], p = 0.037). Combining the datasets resulted in a similar overall effect, reduced CI and increased significance when treated with 2 µM RI-BMAP-28 (combined d = 6.31, 95% CI [1.11–11.52], p = 0.0003 vs original d = 6.88, 95% CI [−2.72–16.48], p = 0.0023). Combining the datasets decreased the overall effect and the CI and increased the significance when treated with 2 µM D-BMAP-28 (combined d = 16.67, 95% CI [13.98–19.36], p = 0.0000017 vs original d = 37.8, 95% CI [35.80–39.80], p = 0.0001). Promastigote viability was not significantly modified when treated with 0.5 µM L-, RI- and D-BMAP-28.

The raw data from the viability assays are available as S1 Data and at the Open Science Framework: osf.io/ushjy.

## Discussion

We sought to independently validate the reported *in vitro* anti-leishmanial effect of L-BMAP-28, D-BMAP-28 and RI-BMAP-28 [5]. We confirmed that BMAP-28 isomers demonstrate anti-leishmanial activities against *L.* major promastigotes *in vitro* with the D- and RI- forms showing more potency than the L- isomer. However, in this replication attempt, the effects at 0.5 µM and 2 µM concentrations were not as large as those originally reported [5]. Previous studies have demonstrated that the L- and D- forms of BMAP-28 can be toxic to mammalian epithelial cells and monocytes with almost 100% toxicity at 6 µM [9]. The RI form showed limited toxicity in this study [9]. These data should be taken into perspective in deciding whether these peptides warrant further experimentation in cell studies or beyond, including testing in humans.

This replication additionally identified that amidated BMAP-28-NH_2_ peptides are more potent anti-leishmanials than unmodified peptides. This effect is consistent with previous studies that have shown amidated peptides to be significantly more active than non-modified peptides [10].

The meta-analytic approach used to combine the original and replication datasets in this study has increased the precision of the effect estimate, with the ‘real’ effect more likely to be represented by the meta-analysis than by the original or the replication studies separately. This approach emphasizes the importance of replication studies to strengthen estimates of effect size for important novel data presented from research studies.

## Supporting Information

S1 Data **Raw data from promastigote viability assays.** Raw fluorescence readings from each of the three biological replicate experiments are provided, along with combined data from background fluorescence corrected averages and combined normalized data (shown as a percentage of the untreated control).
(XLSX)
